# Specific inhibition by synthetic analogs of pyruvate reveals that the pyruvate dehydrogenase reaction is essential for metabolism and viability of glioblastoma cells

**DOI:** 10.18632/oncotarget.5486

**Published:** 2015-10-15

**Authors:** Victoria I. Bunik, Artem Artiukhov, Alexey Kazantsev, Renata Goncalves, Danilo Daloso, Henry Oppermann, Elena Kulakovskaya, Nikolay Lukashev, Alisdair Fernie, Martin Brand, Frank Gaunitz

**Affiliations:** ^1^ A.N. Belozersky Institute of Physicochemical Biology, Lomonosov Moscow State University, 119234 Moscow, Russia; ^2^ Faculty of Bioengineering and Bioinformatics, Lomonosov Moscow State University, 119234 Moscow, Russia; ^3^ Faculty of Chemistry, Lomonosov Moscow State University, 119234 Moscow, Russia; ^4^ Buck Institute for Research on Aging, 8001 Redwood Blvd, Novato, CA 94945, USA; ^5^ Max-Planck-Institute of Molecular Plant Physiology, 14476 Potsdam-Golm, Germany; ^6^ Department of Neurosurgery, Medical Faculty of the University of Leipzig, 04103 Leipzig, Germany; ^7^ Faculty of Biology, Lomonosov Moscow State University, 119234 Moscow, Russia

**Keywords:** pyruvate dehydrogenase, pyruvate synthetic analog, acetyl phosphinate, acetyl phosphonate, glioblastoma viability

## Abstract

The pyruvate dehydrogenase complex (PDHC) and its phosphorylation are considered essential for oncotransformation, but it is unclear whether cancer cells require PDHC to be functional or silenced. We used specific inhibition of PDHC by synthetic structural analogs of pyruvate to resolve this question. With isolated and intramitochondrial PDHC, acetyl phosphinate (AcPH, K_i_^AcPH^ = 0.1 μM) was a much more potent competitive inhibitor than the methyl ester of acetyl phosphonate (AcPMe, K_i_^AcPMe^ = 40 μM). When preincubated with the complex, AcPH also irreversibly inactivated PDHC. Pyruvate prevented, but did not reverse the inactivation. The pyruvate analogs did not significantly inhibit other 2-oxo acid dehydrogenases. Different cell lines were exposed to the inhibitors and a membrane-permeable precursor of AcPMe, dimethyl acetyl phosphonate, which did not inhibit isolated PDHC. Using an ATP-based assay, dependence of cellular viability on the concentration of the pyruvate analogs was followed. The highest toxicity of the membrane-permeable precursor suggested that the cellular action of charged AcPH and AcPMe requires monocarboxylate transporters. The relevant cell-specific transcripts extracted from Gene Expression Omnibus database indicated that cell lines with higher expression of monocarboxylate transporters and PDHC components were more sensitive to the PDHC inhibitors. Prior to a detectable antiproliferative action, AcPH significantly changed metabolic profiles of the investigated glioblastoma cell lines. We conclude that catalytic transformation of pyruvate by pyruvate dehydrogenase is essential for the metabolism and viability of glioblastoma cell lines, although metabolic heterogeneity causes different cellular sensitivities and/or abilities to cope with PDHC inhibition.

## INTRODUCTION

**Figure 1:**
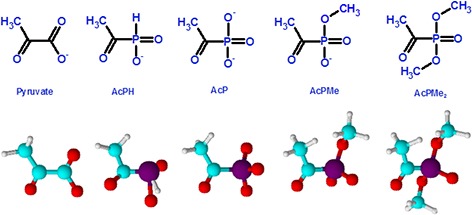
Structures of pyruvate and its synthetic analogs with the phosphinate or phosphonate group substituting for the leaving carboxyl group The anions existing at physiological pH are shown. AcPH, acetyl phosphinate; AcP, acetyl phosphonate; AcPMe, methyl ester of acetyl phosphonate; AcPMe_2_, dimethyl ester of acetyl phosphonate.

Functioning at a branch point of metabolism, the thiamin diphosphate (ThDP)-dependent pyruvate dehydrogenase is an important target for metabolic regulation. Synthetic analogs of 2-oxo acids with a phosphonate or phosphinate group substituting for the leaving carboxyl group (*P*-analogs, Fig. 1), are potent inhibitors of (ThDP)-dependent dehydrogenases [1]. The high inhibitory power and selectivity of the *P*-analogs of 2-oxo acids to their cognate ThDP-dependent dehydrogenases are due to the formation of tightly bound transition state analog complexes after adduction of the *P*-analogs to the active site ThDP. Given the highly specific structure of enzymatic transition states, which differ also in the mechanistically similar ThDP-dependent 2-oxo acid dehydrogenases and decarboxylases, the binding of *P*-analogs is able to discriminate even close family members. The *P*-analogs of 2-oxoglutarate or pyruvate have also been shown to have orders of magnitude lower affinities to non-cognate dehydrogenases or non-ThDP-dependent enzymes (reviewed in [1]). Hence, selective inhibition of pyruvate dehydrogenase (PDH) *in vivo* may be achieved using *P*-analogs of pyruvate. Nevertheless, the potential of the *P*-analogs of pyruvate for directed metabolic regulation has not been systematically evaluated (reviewed in [1]).

Here, we use the *P*-analogs to resolve the controversy regarding the functional state of PDH in cancer cells. According to multiple reports, the PDH complex (PDHC) and its regulation by phosphorylation are important for oncotransformation [1–5]. However, it is unclear whether cancer cell proliferation requires PDH function or is, in contrast, associated with PDH inactivation. A non-functional phosphorylated PDH is a generally inferred hallmark of transformation [2, 3]. Indeed, increased expression of PDH kinases, which inactivate PDH by phosphorylation, has been reported in cancer cells, and is supposed to underlie mitochondrial dysfunction and the Warburg effect [2, 3]. In good accordance with this view, inhibition of PDH kinases by dichloroacetate [6] or other inhibitors [7] activated mitochondrial function and decreased proliferation of cancer cells. However, in other cases, activation of PDH kinases, leading to increased phosphorylation and inactivation of PDH, also impaired proliferation of cancer cells [5, 8]. Finally, highly active PDH was observed in some tumors [9, 10].

To resolve the question of the role of PDH function in cancer metabolism, the direct and selective inhibition of PDH by *P*-analogs of pyruvate provides advantages over the indirect action of PDH kinase effectors. Asides from the possibility that the PDH kinase effectors may also bind to other kinases, PDH kinase itself may have targets other than PDH, especially in cancer cells. Besides, interference with regulation of biological systems often shows non-monotonous dependence [11]; therefore the action of kinase effectors or of changed kinase expression may depend on the original level of expression of PDH kinases and phosphatases. All these factors may differ in metabolically heterogeneous cancer cells compared to non-transformed ones, leading to unsafe conclusions regarding the functional state of PDH in cancer, discussed above.

In this work, we characterized the action of *P*-analogs of pyruvate (Fig. 1) on mammalian systems: partially isolated enzymes (*in vitro*), mitochondria, and cell lines in culture (*in situ*). Comparative analysis showed that the phosphinate analog had a higher potential to inhibit mammalian PDH, either isolated or inside mitochondria, than the phosphonate analog. Although the negative charge of the *P*-analogs is essential for PDH inhibition, the membrane permeability of the uncharged precursor strongly increased the inhibitory power in cultured cells. The impact of the inhibitors on metabolism and viability of different cell lines was studied using metabolic profiling and cellular ATP assays. The action of *P*-analogs of pyruvate revealed that PDH function is essential for viability of different cell lines, including those of human embryonic kidney (HEK293) and highly malignant glioblastomas. The results expose the *P*-analogs of pyruvate as promising tools to reveal the metabolic impact of the PDH reaction in different cells and/or metabolic settings. The identification of cell-specific vulnerability to perturbation in the PDH metabolic checkpoint should provide important information for appropriate tailoring of antiproliferative treatments taking into account the metabolic heterogeneity of cancer cells.

## RESULTS

### Study of the synthetic pyruvate analogs *in vitro*

**Figure 2:**
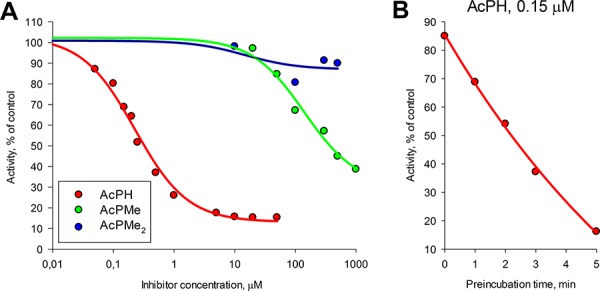
Inhibitory action of AcPH, AcPMe and AcPMe2 on isolated PDHC The reaction was started with 2 mM pyruvate after PDHC from rat heart had been preincubated at 37°C in 50 mM MOPS buffer (pH 7.6) containing 1 mM MgCl_2_, 1 mM CaCl_2_, 1 mM ThDP, 0.05 mM CoA, 2.5 mM NAD^+^, 1 mM DTT and indicated inhibitor concentrations. **A.** Concentration dependences of PDHC activity on the indicated inhibitors after 1 min preincubation. Non-linear regression to a hyperbolic function $\left(y=y_{0}+\frac{ab}{b+x}\right)$ was made using SigmaPlot 12.0. **B.** Time-dependent inhibition of the overall PDHC activity by AcPH. PDHC was preincubated as described above with 0.15 μM AcPH, followed by the reaction start with 2 mM pyruvate at the indicated times. Velocities were measured from the linear part of the product accumulation curves during 0.5–3.5 min of the reaction. Inhibition is presented as % of control activity in the absence of AcPH. Non-linear regression to an exponential decay function (*y*=*y*_0_*e^−kx^*) was made using SigmaPlot 12.0.

The inhibition of isolated PDHC from rat heart by different concentrations of acetyl phosphinate (AcPH) or the mono- (AcPMe) or di- (AcPMe_2_) methyl esters of acetyl phosphonate (Fig. 1) is shown in Fig. 2A. The rate of the overall PDHC reaction was measured after a short (1 min) preincubation of the enzyme with these *P*-analogs of pyruvate. The concentration of pyruvate (2 mM) was saturating, as indicated by published values of K_m_^Pyr^ = 0.02–0.14 mM for heart PDHC under a variety of conditions [12–14]. AcPH strongly inhibited PDHC in the 10^−7^–10^−6^ M concentration range (IC_50_~0.2 μM). Despite possessing the same charge (−1), AcPMe was substantially less inhibitory (IC_50_~0.6 mM under identical conditions), and AcPMe_2_, which is uncharged, had little effect (Fig. 2A). Increasing the preincubation time with AcPH led to a further decrease in activity (Fig. 2B). Although preincubation as such did not affect PDHC, after 5 min preincubation with 0.15 μM AcPH more than 80% of the PDHC activity disappeared, as assayed at 2 mM pyruvate. This further decreased IC_50_ (to less than 0.1 μM, not shown). The time-dependent inactivation was much more pronounced with AcPH compared to AcPMe and AcPMe_2_. That is, under otherwise identical conditions, after 5 min of PDHC preincubation in the potassium phosphate buffer, pH 7.6, the assay at 2 mM pyruvate revealed more than 90% activity loss in the presence of 0.5 μM AcPH, whereas 50 μM AcPMe and AcPMe_2_ decreased activity by only approximately 30% and 10%, respectively (data not shown).

**Figure 3:**
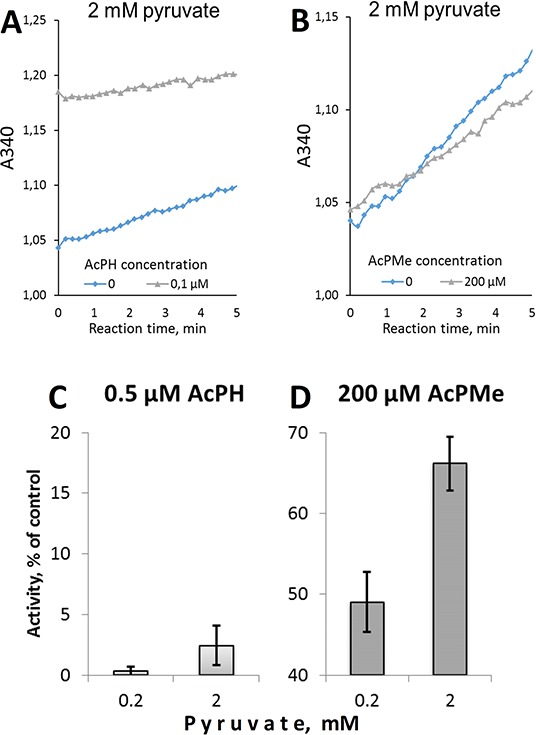
Irreversibility of PDHC inhibition upon preincubation with AcPH and AcPMe Assays were performed at 37°C in 30 mM potassium phosphate (pH 7.0) containing 3 mM NAD^+^, 0.05 mM CoA, 2 mM DTT, 0.4 mM ThDP, 2 mM MgCl_2_ and indicated concentrations of pyruvate and inhibitors. **A, B.** Representative product accumulation curves in the presence or absence of the pyruvate *P*-analogs. Reactions were started by addition of 2 mM pyruvate after PDHC was preincubated for 5 min at 37°C in pyruvate-free assay medium without (grey triangles) or with (blue diamonds) the inhibitors indicated on the figures. **C, D.** Dependence of inhibition by AcPH or AcPMe on pyruvate added after 5 min preincubation with the inhibitors.

The inactivation by AcPH in the preincubation medium was not reversed by pyruvate during the subsequent assay. This is seen from the lack of a detectable lag-period in the product accumulation curves after pyruvate addition to AcPH-inactivated PDHC (Fig. 3A). Linearity of the product accumulation curves in the presence of AcPH or AcPMe (Figures 3A, 3B) also indicates that no additional inactivation occurred with either of the *P*-analogs after pyruvate was added to the inhibitor-containing medium. Thus, pyruvate protects PDHC from further inactivation by AcPH in the reaction medium (no decrease in the reaction rate during the assay in Fig. 3A), but cannot restore the activity already lost during the preincubation with AcPH (no lag-period in Fig. 3A). As a result, only negligible activity was assayed after preincubation with AcPH, independent of the pyruvate concentration in the reaction medium (Fig. 3C). In contrast, competition with pyruvate was seen after preincubation with AcPMe (Fig. 3D), confirming that the inhibition is largely reversible in this case. Thus, when the irreversible process does not dominate the decrease in PDH activity in the presence of the *P*-analogs, their competition with pyruvate at the active site is seen. However, under the experimental set-up when competition kinetics is compromised by an irreversible loss of PDHC activity, as occurs upon preincubation with AcPH, a 10-fold difference in the pyruvate concentration does not significantly affect the final level of inhibition (Fig. 3C).

No significant tissue-specific differences in the action of AcPH or AcPMe were revealed when PDHC from liver or heart were tested, in good accordance with the mechanism of action of the inhibitors, dependent on the universal catalytic mechanism of the 2-oxo acid dehydrogenases. We also tested the *P*-analogs of pyruvate on the other members of the protein family, the 2-oxoglutarate dehydrogenase and branched-chain 2-oxo acid dehydrogenase complexes (OGDHC and BCODHC), which were isolated from liver. Preincubation of the OGDHC or BCODHC with 0.05 mM AcPH, which inhibited PDHC more than 80% at saturating pyruvate (Fig. 2A), did not significantly inhibit the OGDHC or BCODHC. Even at half-saturation with 2-oxoglutarate, i.e. at 0.2 mM 2-oxoglutarate, which is comparable to the enzyme K_m_^OG^ = 0.1–0.2 mM [15–17], OGDHC was not inhibited by 0.05 mM AcPH, and increasing AcPH to 0.2 mM inhibited OGDHC by only 20%. At 0.5 mM 3-methyl-2-oxovalerate (K_m_ ~ 0.02 mM [18]), BCODHC was inhibited by approximately 20% at 0.05–0.2 mM AcPH. These results are consistent with a high degree of AcPH specificity in inhibiting the cognate PDHC. AcPMe did not inhibit OGDHC or BCODHC under the same conditions.

### Action of the synthetic pyruvate analogs in alamethicin-permeabilized mitochondria

**Figure 4:**
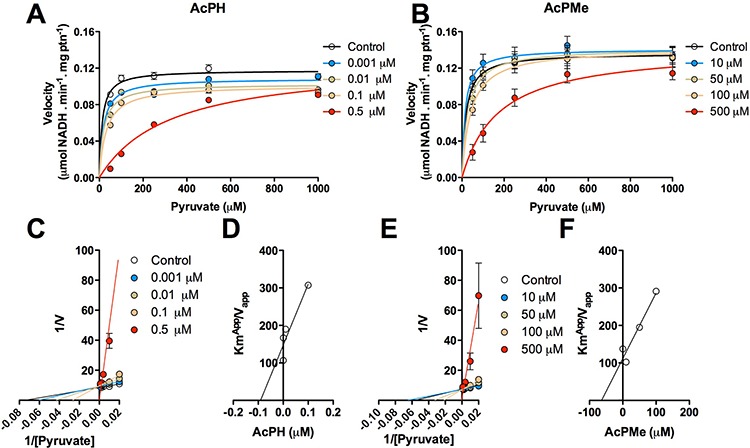
Kinetics of inhibition of PDHC by AcPH and AcPMe in alamethicin-permeabilized mitochondria from rat skeletal muscle The reaction was started by addition of mitochondria. Michaelis-Menten plots show the dependence of PDHC activity on pyruvate (50, 100, 250, 500, 1000 μM) at different concentrations of AcPH **(A)** and AcPMe **(B)** Each inhibitor was loaded into separate 96 well plates. Endogenous calibration curves to measure the NADH reduction in each of the plates were used. Variation in the control V_max_ values between different plates, preparations and/or permeabilization of mitochondria was insignificant (within 10%). Double-reciprocal plots of the data presented in (A) and (B) reveal competition between pyruvate and AcPH **(C)** or AcPMe **(E).** for binding to PDHC. Secondary inhibition plots were built to determine K_i_ for AcPH **(D)** and AcPMe **(F).**

To better understand the action of AcPH and AcPMe in complex biological systems, where, in particular, no preincubation of PDHC with analogs is possible due to the permanent presence of pyruvate, the PDHC activity was assayed in alamethicin-permeabilized mitochondria. In this set of experiments, inhibitors and substrate were presented simultaneously, and the initial rates were recorded, to allow analysis of the PDHC interaction with inhibitors when irreversible inactivation was not promoted (Fig. 4). Pyruvate (0.05–1 mM) was titrated at different concentrations of AcPH (0.001–0.5 μM) or AcPMe (10–50 μM) (Figures 4A, 4B). The reciprocal plots showed that both inhibitors are competitive with pyruvate, since they increased K_m_^Pyr^ without changing maximal velocity (Figures 4C, 4E). Inhibition constants (K_i_) were calculated by plotting K_m_^app^/V_max_^app^ at several values of AcPH and AcPMe against inhibitor concentration. The kinetic parameters obtained in Fig. 4 are summarized in Table 1. As expected from the data obtained with the partially isolated PDHC, AcPH was more potent than AcPMe, K_i_ values were 0.1 μM and 40 μM, respectively (Figures 4D, 4F). In accordance with the *in vitro* data, the *P*-analogs of pyruvate also failed to inhibit activity of the OGDHC in isolated permeabilized mitochondria (data not shown).

**Table 1:** Kinetic analysis of inhibition by AcPH and AcPMe of intramitochondrial PDHC without preincubation with the analogs

AcPH		AcPMe	
V^max^ (μmol NADH/(min × mg protein))	K_i_ (μM)	V^max^ (μmol NADH/(min × mg protein))	K_i_ (μM)
0.1	0.1	0.13	40
Inhibitor concentration (μM)	K_m_^app^ (μM)	Inhibitor concentration (μM)	K_m_^app^ (μM)
0	12.5	0	18.7
0.001	18.1	10	14.4
0.01	19.4	50	27.5
0.1	31.0	100	41.1
0.5	324.2	500	173.4

V_max_ and K_m_ data were obtained by non-linear regression of the experimental dependences presented in Figures 4A, 4B, to the Michaelis-Menten equation using Prism 5.0 software (Graphpad). K_i_ is the x-axis intercept when plotting apparent K_m_ (K_m_^app^) obtained at different inhibitor concentrations divided by V^app^ against AcPH and AcPMe concentrations (Figure 4F).

### Action of synthetic pyruvate analogs in cell cultures

**Figure 5:**
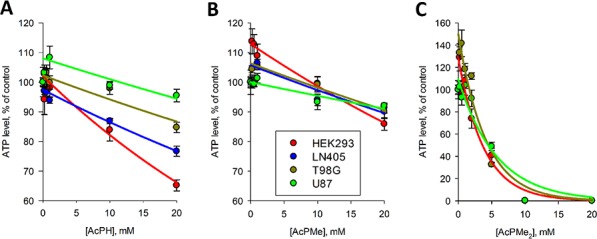
Influence of AcPH (A) AcPMe (B) and AcPMe2 (C) on total ATP levels of different cell cultures Cells were incubated with the indicated inhibitors for 5 h in HBSS. The color code and cell lines tested are shown on the graphs in order of decreased cellular sensitivity to the inhibitors. Non-linear regression to an exponential equation (*y*=*y*_0_*e^−kx^*) was made by SigmaPlot 12.0, with the regression parameters presented in Table 2. When observed, an increase in ATP levels in response to low concentrations of the inhibitors was excluded from the non-linear regression, which was justified by better correlation coefficients R^2^.

The influence of the *P*-analogs of pyruvate on the viability of cultured cells, measured by the luciferase assay of cellular ATP levels, is shown in Fig. 5. The parameters for the ATP decreases after a fixed (5 h) preincubation time with different concentrations of *P*-analogs are presented in Table 2. At lower concentrations of the inhibitors, a slight increase in ATP levels or a delay in the ATP response were observed. Although these deviations were rarely statistically significant, the correlation coefficients of non-linear regression analysis (R^2^, Table 2) improved when the increase or lag phase were omitted from the regression. In these cases, the extrapolated *y*_0_ values of regression curves could deviate from 100% (Fig. 5), with the increases in *y*_0_ up to 150% (Table 2) supporting a complex kinetics of the initial response of cellular ATP levels to the PDH inhibitors. Remarkably, the difference between the inhibitory power of AcPH and AcPMe upon cellular incubation with the *P*-analogs (Fig. 5, Table 2) was minor compared to that observed *in vitro* (Figures 2, 3) and in permeabilized mitochondria (Fig. 4, Table 1). Moreover, all cells were strongly impaired by the uncharged AcPMe_2_ (Fig. 5, Table 2), which was inactive on the isolated enzyme (Fig. 2A). Thus, cellular permeability of the charged *P*-analogs AcPH and AcPMe could limit their intracellular inhibition of PDHC, while uncharged membrane-permeable AcPMe_2_, which could be de-esterified by intracellular enzymes, is highly effective in cell culture.

**Table 2:** Parameters of the regression of experimental ATP decrease in cells incubated with AcPMe, AcPH and AcPMe_2_ to the exponential equation *y*=*y*_0_*e^−kx^*

Inhibitors	AcPMe			AcPH			AcPMe_2_		
Cells	y_0_	k	R^2^	y_0_	k	R^2^	y_0_	k	R^2^
HEK293	113	0.013	0.984	102	0.022	0.990	128	0.261	0.985
LN405	106	0.008	0.876	97	0.012	0.966	-	-	-
T98G	106	0.008	0.985	103	0.008	0.880	150	0.244	0.957
U87	100	0.005	0.882	108	0.007	0.926	109	0.175	0.937

The approximated curves and conditions are given in Figure 5.

It is also obvious from Figures 5A, 5B and regression constants in Table 2 that the PDHC inhibitors AcPH and AcPMe affected ATP levels more strongly in HEK293 cell cultures than in glioblastoma cell lines. As seen from values of *k* in Table 2, the difference was especially obvious when HEK293 and U87 cell lines were compared, and persisted also when the membrane-permeable AcPMe_2_ was applied. The cell-specific sensitivity to the *P*-analogs of pyruvate exposes the varied metabolic impact of PDHC inhibition on viability of different cell lines.

### Action of AcPH on the cell metabolome

**Figure 6:**
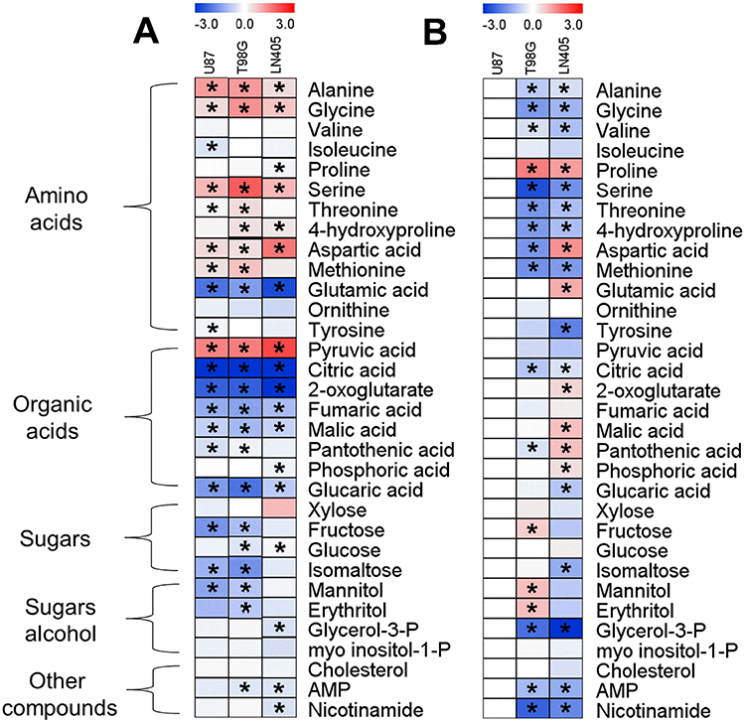
Comparative metabolic profiling of glioblastoma cell lines treated with 0.5 mM AcPH for 5.5 h (A) and under control conditions (B) **A.** Fold changes in the metabolite levels of AcPH-treated T98G, U87 or LN405 cell lines, compared to untreated controls. **B.** Metabolic difference between untreated cell lines is presented as the fold changes in metabolite levels of the T98G or LN405 lines, compared to the U87 line.

In the metabolomics study, we aimed to detect the primary action of PDHC inhibition on the cellular metabolome. Therefore, we exposed cells to a low concentration of AcPH, which was chosen as the best structural analog of pyruvate (Fig. 1), inhibiting PDH at 10^−7^ M concentrations (Figures 2, 3) and directly, i.e. not requiring intracellular activation. These features of AcPH were advantageous for reproducible metabolomics analysis of the primary changes, because interpretation of the action of a stronger inhibitor of cellular viability AcPMe_2_ would be complicated by the time- and concentration-dependent intracellular formation of multiple inhibitory species (AcPMe and fully de-esterified acetyl phosphonate (AcP), Fig. 1) from the precursor. As seen from Fig. 6A, the incubation with 0.5 mM AcPH for 5.5 h significantly changed many cellular metabolites in all glioblastoma cell lines, despite no detectable changes in an ATP-based viability assay (Fig. 5A). Obviously, the viability decrease results from multiple primary and secondary metabolic changes, while the metabolomics changes reflect initial perturbation due to PDHC inhibition. As expected, pyruvate and amino acids that are degraded through pyruvate, i.e. Ala, Gly, Ser, Thr, were strongly accumulated, whereas the TCA cycle intermediate citrate for which the PDHC product acetyl-CoA is a precursor, and the citrate transformation product 2-oxoglutarate were strongly decreased. The lower level of 2-oxoglutarate was coupled to a strong decrease in glutamate. The levels of fumarate and malate were significantly decreased, while aspartate was significantly increased in all cell lines studied. Several other amino acids, organic acids, sugars, sugar alcohols, AMP and nicotinamide changed in response to AcPH (Fig. 6A). However, unlike the predictable consequences of PDHC inhibition, these changes seem to be more dependent on cell-specific metabolism. To compare the metabolic profiles of the non-treated cell lines, the levels of metabolites in the two cell lines (T98G and LN405) were related to those in the third one (U87) used as a reference. The resulting heat map presented in Fig. 6B exposes the cell-specific differences in the steady-state levels of the detected metabolites. For instance, lower levels (blue scale) of fructose, isomaltose, mannitol and erythritol are observed in the non-treated LN405 cells, compared to the non-treated U87 and T98G cells (Fig. 6B). In the cells treated with AcPH, these metabolites strongly decrease in U87 and T98G cell lines, but are not responsive to the treatment in LN405 cell line (Fig. 6A). Thus, original differences in cellular metabolism cause cell-specific responses to AcPH. As a result, comparative metabolomics indicates that all cell lines show similar response to AcPH of pyruvate and its metabolic partners linked through the TCA cycle (metabolites in the upper part of Fig. 6A). However, extension of these perturbations to other pathways, such as those defining the levels of sugars and nucleotides (metabolites in the lower part of Fig. 6A), occurs according to the cell-specific metabolism, expressed in different metabolic profiles of the non-treated cells (Fig. 6B).

### Analysis of transcriptomics data

For three out of four cell lines tested with the PDHC inhibitors in our experiments, the global Affymetrix gene expression levels are available from the Gene Expression Omnibus (GEO) database. It was therefore interesting to compare cellular sensitivity to the PDHC inhibitors with the expression of relevant genes. Signal intensities of annotated genes of interest, extracted from the database, are presented in Table 3. Apart from the well-known genes for the PDHC components (*PDHA1, PDHB, DLAT, PDHX, DLD*) and its phosphorylation system (pyruvate dehydrogenase kinases *PDK1–3* and phosphatases *PDP1–2*) shown in Table 3, we included in the analysis a testis-specific isoform of PDHA, *PDHA2*, a regulatory subunit of the pyruvate dehydrogenase phosphatase, *PDPR*, and *PDK4*. However, none of the cell cultures expressed significant levels of mRNAs for *PDHA2, PDPR* and *PDK4*, owing to which they were not included in Table 3. Compared to the HEK293 cell line, glioblastoma cell lines U87 and T98G are characterized by significantly lower expression of the genes for the protein components and phosphorylation system of PDHC (Table 3). Nevertheless, the ratio of the first (*PDHA1*+*PDHB*) and second (*DLAT*) components of PDHC is similar in HEK293 and T98G lines, favoring the catalytic competence of PDHC in both lines. In contrast, a detectable signal of mRNA for the second complex component *DLAT* was repeatedly absent in different analyses of the U87 line (Table 3), suggesting that the overall PDHC reaction, which requires all the complex components, is impaired in U87 cells.

**Table 3:** Transcriptomics data on the components of PDHC and selected monocarboxylate transporters in the cell lines used in this study

Category	Cell line \ mRNA	HEK293 (11)	T98G (7)		U87 (11)		
		median ± SEM	median ± SEM	ttest (with HEK293)	median ± SEM	ttest (with HEK293)	ttest (with T98G)
**PDHC catalytic and assembly components**	***PDHA1***	0.472 ± 0.050	0.050 ± 0.041	0.0003	0.051 ± 0.032	0.0001	0.4780
	***PDHB***	0.148 ± 0.014	0.024 ± 0.013	0.0000	0.043 ± 0.018	0.0001	0.0823
	***DLAT***	0.157 ± 0.032	0.014 ± 0.011	0.0055	0.000 ± 0.001	0.0001	0.0002
	***DLD***	0.241 ± 0.024	0.148 ± 0.054	0.0035	0.067 ± 0.021	0.0000	0.0000
	***PDHX***	0.131 ± 0.011	0.059 ± 0.024	0.0003	0.023 ± 0.007	0.0000	0.0006
**PDHC regulatory components**	***PDK1***	0.009 ± 0.010	0.008 ± 0.004	0.0984	0.002 ± 0.004	0.1030	0.3568
	***PDK2***	0.024 ± 0.006	0.004 ± 0.003	0.0236	0.000 ± 0.001	0.0013	0.0734
	***PDK3***	0.015 ± 0.003	0.000 ± 0.001	0.0043	0.001 ± 0.000	0.0005	0.4215
	***PDP1***	0.019 ± 0.005	0.036 ± 0.011	0.2346	0.008 ± 0.005	0.0796	0.0849
	***PDP2***	0.014 ± 0.002	0.000 ± 0.000	0.0004	0.001 ± 0.000	0.0000	0.1580
**Pyruvate and / or lactate carriers**	***SLC16A1***	0.384 ± 0.028	0.042 ± 0.035	0.0000	0.020 ± 0.011	0.0000	0.0375
	***SLC16A7***	0.012 ± 0.003	0.012 ± 0.009	0.1714	0.001 ± 0.001	0.0086	0.0156
	***SLC16A8***	0.000 ± 0.000	0.087 ± 0.043	0.0001	0.166 ± 0.045	0.0001	0.2213
	***SLC16A3***	0.000 ± 0.000	0.000 ± 0.000	0.0331	0.001 ± 0.000	0.0032	0.3387
	***MPC1***	0.174 ± 0.018	0.039 ± 0.016	0.0000	0.051 ± 0.018	0.0001	0.2724
	***MPC2***	0.164 ± 0.025	0.067 ± 0.037	0.0198	0.209 ± 0.053	0.3819	0.0327

The relative expression levels of mRNAs of the annotated genes of interest were extracted from global transcriptomics data deposited in the GEO database. The numbers of experiments used for the calculations of median and SEM values are shown in parentheses.

Because AcPH and AcPMe (Fig. 1) are structural analogs of pyruvate carrying the same charge (−1), they may use and/or block the pyruvate carriers. Besides, cellular resistance to PDHC inhibition may be affected by the ability to extrude lactate which accumulates along with pyruvate due to lactate dehydrogenase-catalyzed reduction of pyruvate. Relative expression of the genes for relevant monocarboxylate transporters and parameters of their substrate specificity according to [19] are presented in Tables 3 and 4, respectively. The expression data (Table 3) show that signals of mRNA for *SLC16A1* (*MCT1*), coding for the major cellular monocarboxylate transporter, and the two subunits of the hetero-oligomeric pyruvate transporter of the inner mitochondrial membrane, *MPC1* and *MPC2*, are proportional to those of the rate-limiting component of PDHC, (*PDHA1*+*PDHB*). The protein product of the *SLC16A7* (*MCT2*) gene is a minor carrier which is, however, very specific for pyruvate, preferring it over lactate (Table 4). Expression of *SLC16A7* is similar in HEK293 and T98G but much lower in U87 cells (Table 3). Expression of highly-specific lactate transporters *SLC16A8* (*MCT3*) and *SLC16A3* (*MCT4*), which prefer lactate over pyruvate (Table 4), is pronounced in glioblastoma cell lines T98G and U87, but not detectable in HEK293 cells (Table 3).

**Table 4:** Substrate specificity of some SLC16 family members

Gene (Alt. name)	K_m_^pyruvate^ (mM)	K_m_^lactate^ (mM)	K_m_^2-hydroxybutyrate^ (mM)	K_m_^acetoacetate^ (mM)
SLC16A1 (MCT1)	0.7	4.5	2.6	5.5
SLC16A7 (MCT2)	0.08	0.74	ND	0.8
SLC16A8 (MCT3)	ND	6	ND	ND
SLC16A3 (MCT4)	153	28	56	216

K_m_ values for pyruvate, L-lactate, D,L-2-hydroxybutyrate and acetoacetate are given from [19]. ND – no detectable transport was observed.

## DISCUSSION

### Mechanism of P-analog inhibition of mammalian PDHC

Inhibition of PDHC by *P*-analogs of pyruvate has been studied using bacterial and plant enzymes [20–24]. In the present work, using mammalian PDHC from different tissues, we reveal that certain features of the mechanism of action and structure-function relationship of the inhibitors are common for pyruvate dehydrogenases from all clades. First, the analogs compete with pyruvate binding at the active site (Fig. 4), and the phosphinate analog (AcPH) is orders of magnitude more potent than the phosphonate analog with the same charge -1 (AcPMe) (Fig. 2A, Fig. 4 and Table 1). The difference may be due to lower steric hindrance for the ThDP reaction with the carbonyl group near the phosphinate residue compared to the phosphonate, and a higher effective positive charge on the phosphorus in phosphinates than phosphonates [1]. Second, the non-charged dimethylated phosphonate analog, AcPMe_2_, inhibits poorly *in vitro* (Fig. 2A). Thus, to mimic pyruvate binding to PDHC, analogs need a negative charge. However, similar to the phosphonate analogs of 2-oxoglutarate [25, 26], the non-charged AcPMe_2_ is active in cells (Fig. 5C). Obviously, intracellular activation of this precursor by esterases forms the charged inhibitory species AcPMe (charge -1) and AcP (charge -2) (Fig. 1).

Dependence of the maximal inhibitory effect of the most potent inhibitor, AcPH, on its preincubation with PDHC (Fig. 2B) is the third feature of *P*-analog inhibition, common for mammalian, plant and bacterial systems. Studies on bacterial PDHC showed that during preincubation the first inhibitory complex between AcPH and PDH undergoes a transformation, resulting in a slowly dissociating, yet fully reversible, binding of the inhibitor to PDH [20, 24]. In contrast, the slow dissociation of AcPH does not occur in mammalian PDH, since there is no slow reactivation in reaction medium, indicating that excess pyruvate fails to restore the activity lost during preincubation with AcPH (Figures 2B, 3C). Only when pyruvate is present during phosphonate binding is PDHC protected from inactivation by AcPH (Fig. 3A), and only then is AcPH inhibition of PDHC satisfactorily described by reversible competition with pyruvate at the active site of PDH (Fig. 4, Table 1). Thus, irreversible inactivation upon preincubation with AcPH (Figures 2A, 3A, 3C), also noted for commercial PDHC from bovine heart [22], distinguishes mammalian PDHC from the bacterial enzyme [20, 24]. Due to the irreversible inactivation, mammalian PDHC is much more sensitive to AcPH (nearly complete inhibition after 5 min at 10^−7^ M, Figures 2B, 3C) than the bacterial enzyme (only 50% inhibition after 10 min at 10^−7^ M [20]). Species-specific irreversible effects of the phosphonate analogs of other 2-oxo acids include dilution-resistant inhibition of plant OGDHC by phosphonate analogs of 2-oxoglutarate [27, 28] and irreversible inactivation of benzoylformate decarboxylase by a phosphonate analog of benzoylformate, where the C-P bond of the bound phosphonate breaks, phosphorylating the active site serine [29]. An analogous mechanism of AcPH-dependent phosphorylation of the active site of PDH may irreversibly inactivate mammalian PDHC, where a serine residue near the pyruvate-binding site is phosphorylated by PDH kinase 1, inactivating PDH. The phosphorylation and pyruvate binding are mutually exclusive [30–33], as are the AcPH-induced inactivation and pyruvate binding (see above). Unlike mammalian PDH, the bacterial enzyme has no phosphorylatable serine residue near the pyruvate binding site and is not inactivated by regulatory phosphorylation. The data may explain the irreversible action of AcPH on mammalian (Figures 2B, 3A, 3C), but not bacterial [20] PDHC, favoring the AcPH-dependent phosphorylation of the mammalian PDH active site.

Earlier *in vitro* tests of interactions of the pyruvate *P*-analogs with other pyruvate-transforming enzymes indicated that binding of *P*-analogs to aminotransferases and lactate dehydrogenase is weak and unlikely to contribute to the action of the analogs at *in vivo* concentrations of pyruvate [1]. Nevertheless, the small size of AcPH and AcPMe could allow their accommodation in the active sites of 2-oxo acid dehydrogenases other than PDH, such as 2-oxoglutarate dehydrogenase and branched-chain 2-oxo acid dehydrogenase, which form tight inhibitory complexes with the *P*-analogs of their specific substrates. However, we showed that, compared to the PDHC inhibition, OGDHC or BCODHC were inhibited insignificantly (≤ 20%) even at orders of magnitude higher concentrations of the *P*-analogs of pyruvate. Thus, our data strongly support selective binding of the *P*-analogs of pyruvate to PDHC *in vivo*.

### Comparison of inhibition by P-analogs *in vitro* and *in situ*

Whereas the IC_50_ of AcPH and AcPMe was about 3000-fold different with partially isolated PDHC even after a short (1 min) preincubation with inhibitors (Fig. 2A), in the pyruvate-containing milieu *in situ* the difference decreased to 400-fold for intramitochondrial PDHC (K_i_, Table 1) and 1.5-fold for intracellular PDHC (k, Table 2). The relative effectiveness of AcPMe_2_ was also different *in vitro* and *in situ*. Able to penetrate the cell membrane without a carrier, uncharged AcPMe_2_ was the strongest inhibitor of cellular viability (Table 2, Fig. 5), although it did not inhibit PDH *in vitro* (Fig. 2A). Similar to other esterified pro-drugs, AcPMe_2_ obviously gives rise to the active charged species after intracellular transformation by esterases. Thus, in addition to the pyruvate-induced protection from the irreversible inactivation of PDHC by AcPH, the similar potency of AcPH and AcPMe in cells is obviously due to limited intracellular delivery of these negatively charged inhibitors. Our analysis of expression of the carriers that transport pyruvate into the cell and the mitochondrial matrix (Tables 3, 4) revealed correlations with the sensitivities to the *P*-analogs of pyruvate. As seen from Table 3, the major transporters of pyruvate into cells (*SLC16A1*) and mitochondria (*MPC1*+*MPC2*) are expressed proportionally to the PDH subunits (*PDHA1*+*PDHB*), suggesting expression to be a measure of the physiologically linked processes of pyruvate influx and degradation. Lower expression of *SLC16A7* in U87 versus HEK293 and T98G (Table 3) agrees with a lower sensitivity of U87 to AcPH and AcPMe, compared to HEK293 and T98G (Fig. 5, Table 2). In view of lactate accumulation upon PDHC inhibition, the ability of cancer cells to extrude lactate faster through higher expression of *SLC16A8*, aided by *SLC16A3* in U87 cells (Table 3), may also contribute to the higher resistance to the *P*-analogs of the glioblastoma vs HEK293 cell lines (Fig. 5, Table 2). Note that our metabolic profiling did not consistently detect lactate in glioblastoma cell lines. The undetectable level of this metabolite, including in cells treated with AcPH, where pyruvate and alanine are accumulated (Fig. 6A), suggests rapid lactate extrusion. As a result, the presence of pyruvate protecting from irreversible inactivation by AcPH, the uptake of the *P*-analogs and lactate export may contribute to the different relative sensitivities of isolated (Fig. 2A) and cellular (Fig. 5) PDHC to AcPH, AcPMe and AcPMe_2_.

### AcPH and AcPMe as tools to understand metabolic transformation in cancer

The functional state of PDHC in cancer is a matter of controversy. On one hand, PDHC is supposed to be down-regulated in cancer due to phosphorylation [2]. This is supported by several findings. For instance, the trans-activation of the gene for PDH kinase isoform 1 (*PDK1*) by HIF-1 is induced in hypoxia [34]; the phosphorylation-induced activation of *PDK1* by oncogenic mitochondrial Tyr kinases is under the control of tumor regulators Myc and HIF-1 [35], and an inhibitor of PDH kinase, dichloroacetate, negatively affects tumor growth, which correlates with PDH activation [6, 36, 37]. However, as mentioned in the Introduction, in other studies, proliferation of cancer cells was impaired concomitant with activation of PDH kinases [5, 8].

Our analysis of expression of PDHC components (Table 3) revealed that highly malignant glioblastoma cells (U87 and T98G) had lower expression of PDHC than immortalized, but benign HEK293 cells, which is in accordance with a lower impact of PDHC inhibitors on cellular viability in U87 and T98G vs HEK293 cells (Fig. 5, Table 2). Remarkably, however, except for *DLAT* in U87 cells, all other catalytic and regulatory components of PDHC as well as pyruvate transporters are expressed at similar ratios in HEK293 and glioblastoma cells (Table 3). The expression suggests a functional significance of PDHC, which agrees with the effects of the PDH inhibitors on the viability indicator ATP and metabolic profiles of the glioblastoma cells (Figures 5, 6). Undetectable expression in U87 cells of the core component of PDHC, *DLAT* (Table 3), suggests impairment in the overall PDHC reaction in these cells, because *DLAT* is required for the complex assembly and *DLAT*-mediated catalysis [38, 39]. Decreased function of PDHC in U87 cells agrees with a lower decrease in U87 viability upon PDHC inhibition (Fig. 5), compared to T98G and HEK293 cells expressing the full set of the PDHC enzymatic components. However, AcPH significantly changes the metabolic profile even in U87 cells (Fig. 6A), and the membrane-permeable AcPMe_2_ strongly decreases the U87 viability (Fig. 5C). These findings suggest that some of the PDHC-catalyzed reactions that do not require *DLAT* and usually are considered as non-physiological side reactions may be important for U87 viability. An example of such a reaction is formation of acetoin, which is catalyzed by PDH, and greatly increases in some tumors [9, 10]. Thus, varied cellular sensitivity to inhibitors of PDHC (Fig. 5) correlates with different cellular expression of PDHC components (Table 3). The metabolic heterogeneity of glioblastoma cells in regard to oxidative decarboxylation of pyruvate is supported by varied transcript levels of the PDHC components and related transporters (Table 3) and different metabolic profiles of these cells (Fig. 6B). For example, significantly lower levels of most of the TCA cycle-degraded amino acids and citrate in T98G cells compared to U87 cells suggest different regulation of the PDHC junction to the TCA cycle in these cells, extended to the differences in metabolism of sugars (fructose, mannitol, erythritol) and other metabolites (AMP, nicotinamide).

The data obtained in this (Figures 5, 6A) and other [40] studies indicate that cell viability, as measured by ATP levels, decreased at much higher concentrations of the inhibitors (Fig. 5) and/or longer inhibition time [40] than needed to induce significant metabolic perturbation (Fig. 6A, [40]). Moreover, metabolic responses of cells to lower concentrations of damaging factors are not adequately reflected by decreases in ATP levels, as such factors may increase ATP levels. When cytotoxic necrotizing factor 1 acted on intestinal cells, ATP increased concomitant with elevation of oxidative phosphorylation [41]. Upon cellular exposure to metabolic inhibitors, a decrease in cellular ATP levels was preceded by a significant increase when OGDHC was inhibited in cultured neurons [40] and in our experiments on PDHC inhibition (Fig. 5, Table 2), which showed simultaneous decreases in AMP levels by metabolic profiling (Fig. 6A). Along with the high (approximately 90%) level of adenine nucleotide phosphorylation in the resting state [42, 43], the observed increases in ATP (Fig. 5), decreases in AMP (Fig. 6A) and disturbed nucleic acid maintenance [40] in the metabolically challenged cells suggest that the ATP increases in the perturbed cells reflect changes in the adenine nucleotide pool size. If changes in cellular ATP levels are indicators of general destabilization of metabolism due to drug-induced metabolic changes, ATP levels may not only decrease, but also increase (Fig. 5) during initial damage.

Our data on the response of different cell lines to PDH inhibitors (Figures 5, 6) clearly show that PDH is not disabled, but important for the viability of glioblastoma cells. Genetic background of the cell-specific metabolism is an important factor potentially contributing to the different cellular sensitivity to PDH inhibitors.

## MATERIALS AND METHODS

### Synthesis of pyruvate analogs

Sodium acetylphosphinate was synthesized according to [21]. Phosphinic acid (50 wt.% in H_2_O) (6.6 ml, 50 mmol) was evaporated at 1 mm Hg at ambient temperature to remove water. Triethyl orthoacetate (18.84 g, 21.3 ml, 116 mmol) was added dropwise under argon. Dry HCl was passed through the solution under vigorous stirring until it became cloudy. The reaction mixture was stirred overnight at ambient temperature under argon, followed by evaporation at 1 mm Hg to remove volatiles. NaOH (3 g, 75 mmol) in 40 ml degassed water was added dropwise at 0°C under argon. The resulting solution was refluxed under argon for 2 h. After cooling, the pH was adjusted to 8.0 with concentrated HCl, and the solution was evaporated to dryness. The residue was triturated with 70 ml of boiling absolute ethanol. After filtration ethanol was evaporated to dryness and the residue was recrystallized from absolute ethanol. The resulting sodium (1,1-diethoxyethyl) phosphinate contained 6 mol% NaH_2_PO_2_ as impurity based on NMR ^1^H and ^31^P spectra: NMR ^1^H (400 MHz, D_2_O), δ, ppm: 7.06 (d, *J* 518.0 Hz, 2H,). NMR ^31^P (161.9 MHz, D_2_O), δ, ppm: 7.1. The yield of sodium (1,1-diethoxyethyl)phosphinate was 22%, m.p. 182–185°C. NMR ^1^H (400 MHz, D_2_O), δ, ppm: 6.89 (d, *J* 515.9 Hz, 1H, HP), 3.74 (q, *J* 7.1 Hz, 4H, OCH_2_CH_3_), 1.41 (d, *J* 11.0 Hz, 3H, CCH_3_), 1.22 (t, *J* 7.1 Hz, 6H, OCH_2_CH_3_); NMR ^13^C (100.6 MHz, D_2_O), δ, ppm: 100.8 (d, *J* 144.1 Hz, CCH_3_), 57.9 (d, *J* 6.8 Hz, OCH_2_CH_3_), 17.4 (d, *J* 11.0 Hz, CCH_3_), 14.6 (OCH_2_CH_3_); NMR ^31^P (161.9 MHz, D_2_O), δ, ppm: 21.5.

Sodium (1,1-diethoxyethyl)phosphinate (1 g, 4.9 mmol) was dissolved in a mixture of 9.5 ml glacial acetic acid and 0.5 ml water. The solution was stirred at ambient temperature for 24 h. The solvent was evaporated to dryness; the residue was triturated with 30 ml of acetone and filtered off to give sodium acetylphosphinate as a white solid. At this step, the yield was 96%, with the final compound containing 6 mol% NaH_2_PO_2_ as impurity (based on NMR ^1^H and ^31^P spectra). NMR spectra showed that a solution of sodium acetylphosphinate in D_2_O contains up to 30% sodium (1,1-dihydroxyethyl)phosphinate (hydrated form): NMR ^1^H (400 MHz, D_2_O), δ, ppm: 6.73 (d, *J* 510.2 Hz, 1H, HP), 1.48 (d, *J* 10.4 Hz, 3H, CCH_3_); NMR ^13^C (100.6 MHz, D_2_O), δ, ppm: 93.7 (d, *J* 140.0 Hz, CCH_3_), 21.6 (d, *J* 13.5 Hz, CCH_3_); NMR ^31^P (161.9 MHz, D_2_O), δ, ppm: 25.4. NMR spectra of the same sample in DMSO-*d*_6_ demonstrate no hydrated form. The following characteristic peaks of sodium acetylphosphinate were observed: NMR ^1^H (400 MHz, D_2_O), δ, ppm: 6.84 (d, *J* 546.5 Hz, 1H, HP), 2.46 (d, *J* 4.3 Hz, 3H, C(O)CH_3_); NMR ^13^C (100.6 MHz, D_2_O), δ, ppm: 223.6 (d, *J* 108.8 Hz, C(O)CH_3_), 27.2 (d, *J* 46.4 Hz, C(O)CH_3_); NMR ^31^P (161.9 MHz, D_2_O), δ, ppm: 12.6. NMR ^1^H (400 MHz, DMSO-*d*_6_), δ, ppm: 6.54 (d, *J* 495.2 Hz, 1H, HP), 2.10 (d, *J* 2.8 Hz, 3H, C(O)CH_3_); NMR ^31^P (161.9 MHz, DMSO-*d*_6_), δ, ppm: 10.6.

O,O'-Dimethyl acetylphosphonate was obtained according to [44]. A mixture of dimethyl phosphite (3.3 g, 2.75 ml, 30 mmol) and ethyl vinyl ether (3.24 g, 4.3 ml, 45 mmol) was added dropwise to acetyl chloride (4.71 g, 4.27 ml, 60 mmol) stirred at 0°C. The mixture was stirred at ambient temperature for 48 h. Products were purified by vacuum distillation. Yield: 3.3 g (72%), b.p. 47–48^°^C/0.9 mm. NMR ^1^H (400 MHz, CDCl_3_), δ, ppm: 3.84 (d, *J* 10.8 Hz, 6H, (CH_3_O)_2_P(O)), 2.46 (d, *J* 5.3 Hz, 3H, C(O)CH_3_); NMR ^31^P (161.9 MHz, CDCl_3_), δ, ppm: −1.0.

O-Methyl sodium acetylphosphonate was prepared according to [45]. To a stirred solution of O,O'-dimethyl acetylphosphonate (1.52 g, 10 mmol) in dry acetone (10 ml) a solution of sodium iodide (11 mmol, 1.65 g) in dry acetone (5 ml) was added dropwise. The reaction mixture was stirred for 18 h at ambient temperature. The precipitate was filtered off, washed with dry acetone (2 ml) and dried in vacuo. Yield: 1.5 g (94%), m.p. 190–191°C. NMR ^1^H (400 MHz, DMSO-*d*_6_), δ, ppm: 3.34 (d, *J* 10.0 Hz, 3H, (CH_3_O)P(O)), 2.15 (d, *J* 3.5 Hz, 3H, C(O)CH_3_); NMR ^13^C (100.6 MHz, D_2_O), δ, ppm: 220.1 (d, *J* 163.6 Hz, C(O)CH_3_), 52.9 (d, *J* 5.9 Hz, (CH_3_O)P(O)), 30.3 (d, *J* 49.7 Hz, C(O)CH_3_); NMR ^31^P (161.9 MHz, DMSO-*d*_6_), δ, ppm: −0.5.

### Enzyme purification

PDHC was partially purified from heart or liver of Wistar rats by modifications of published procedures [46–48]. The tissues were stored frozen at −70°C. Except where indicated, purification was at 4°C, and the pH of the buffers was adjusted at room temperature. The buffers were then cooled to 4°C.

PDHC from heart was isolated using ~8 g of tissue, cut in pieces with scissors and homogenized in 1.5 volumes of isolation buffer A, comprising 0.03 M HEPES, 1 mM EDTA, 0.15 M KCl, 3 mM dithiothreitol (DTT), 1 mM phenylmethanesulfonyl fluoride (PMSF), 1 mM AEBSF, 0.8 μM aprotinin, 50 μM bestatin, 20 μM leupeptin and 10 μM pepstatin pH 7.4. An IKA homogenizer at maximum velocity was used to disperse the tissue, followed by several strokes in a Potter-Elvehjem homogenizer. The homogenate was diluted 2 times with isolation buffer A containing 1% (v/v) Triton X-100 then centrifuged for 20 min at 10,000 g. The pellet was suspended in 5 volumes of isolation buffer A, and the homogenate was centrifuged as above. Combined supernatants were filtered through four layers of cheesecloth, and 35% (w/v) polyethylenglycol (PEG)-6000 solution was added to a final concentration of 1% (w/v). After stirring for 30 min, the suspension was centrifuged for 40 min at 10,000 g. PEG was increased in the supernatant to 4.5% (w/v), and the suspension centrifuged as above. The pellet was dissolved in 1.5 volumes of isolation buffer B, comprising 0.03 M HEPES, 0.1 mM EDTA, 0.1% (v/v) Triton X-100, 0.1 M KCl, 3 mM DTT, 10 mM MgSO_4_, 1 mM AEBSF, 0,8 μM aprotinin, 50 μM bestatin, 20 μM leupeptin and 10 μM pepstatin, pH 7.4, and incubated at 37°C for 20 min. Incubation with magnesium ions allows full activation of PDHC and BCODHC through dephosphorylation. After cooling, the suspension was brought to 3% (w/v) in PEG-6000 and stirred for 30 min, followed by a 40 min centrifugation at 10,000 g. The pellet was resuspended in one volume of isolation buffer C, comprising 0.03 M potassium phosphate buffer, 0.1 mM EDTA, 10% (v/v) glycerol, 0.15 M KCl, 3 mM DTT, 1 mM AEBSF, 0.8 μM aprotinin, 50 μM bestatin, 20 μM leupeptin and 10 μM pepstatin, pH 7.5. PDHC was stored at −20°C for 2 months without significant loss of activity.

OGDHC and BCODHC were also partially purified from rat livers stored frozen at −70°C, by a modification of a published method [48]. Frozen tissue (~40 g) was cut in pieces with scissors and homogenized in one volume of isolation buffer D, comprising 0.05 M MOPS, 2.7 mM EDTA, 1 mM benzamidinium chloride and 1 mM PMSF, pH 7.0, in a Potter-Elvehjem homogenizer. The homogenate was diluted with one volume of isolation buffer D containing 6% (v/v) Triton X-100, and pH was adjusted to 6.8. The homogenate was centrifuged for 20 min at 10,000 g. The pellet was resuspended in one volume of isolation buffer D and centrifuged as above. Combined supernatants were filtered through four layers of cheesecloth and adjusted to pH 6.45 with 10% (v/v) acetic acid followed by addition of 0.12 volumes of 35% (w/v) PEG-6000. After stirring for 30 min, the suspension was centrifuged for 20 min at 18,500 g. The pellet was dissolved in 100 ml isolation buffer D and adjusted to pH 6.8 with 5 M NaOH at 20°C. Insoluble material was removed by centrifugation at 18,500 g for 30 min at 20°C. The supernatant was filtered from fat and left at 4°C overnight. Next morning the supernatant was adjusted to pH 7.0 and 1 M MgCl_2_ was added to a final concentration of 13 mM. The supernatant was incubated on a water bath at 30°C for 5 min. After cooling, cytochalasin D was added to 1 μg/ml. The ionic strength was increased by addition of 1 M potassium dihydrogen phosphate, pH 6.3, to 50 mM. The pH was adjusted to 6.45 with 10% (v/v) acetic acid followed by addition of 0.12 volumes of 35% (w/v) PEG-6000. After stirring for 30 min, the suspension was centrifuged for 30 min at 18,500 g. The resulting pellet was suspended in a minimal volume of isolation buffer E comprising 0.05 M MOPS, 2.7 mM EDTA, 1 mM benzamidinium chloride, 1 mM phenylmethanesulfonyl fluoride (PMSF), 1 mM leupeptin, 20 μM AEBSF and 1% (v/v) Triton X-100, pH 7.0. The suspension was adjusted to pH 6.8 with 5M NaOH at 20°C. Insoluble material was removed by centrifugation at 18,500 g for 40 min and supernatant containing PDHC, OGDHC and BCODHC was stored at −20°C for 3 months without significant loss of OGDHC activity.

Overall reactions of partially purified PDHC, OGDHC and BCODHC were tested spectrophotometrically at 340 nm by NADH production as specified in the figure legends.

### Mitochondrial isolation and permeabilization

5–10 week old female Wistar rats were from Charles River Laboratories fed chow ad libitum and with free access to water. Mitochondria were isolated from hind limb skeletal muscle at 4°C in Chappell-Perry buffer (CP1; 50 mM Tris, 100 mM KCl and 2 mM EGTA, pH 7.4 at 4°C) by standard procedures [49] and kept on ice until used. Protein was measured by the biuret method. The animal protocol was approved by the Buck Institute Animal Care and Use Committee in accordance with IACUC standards. Mitochondria were permeabilized according to [50]. Briefly, intact mitochondria (35 mg mitochondrial protein/ml) were diluted 20-fold in 10 mM HEPES, 0.25 M sucrose, 0.2 mM EDTA, 2.5 mM MgCl_2_, 40 μg/ml alamethicin, and 1 mg/ml fatty acid-free bovine serum albumin, pH 7.4 at 25°C, and incubated for 5 min at room temperature. The suspension was diluted 2.5-fold in the same buffer lacking MgCl_2_, alamethicin and albumin, then centrifuged at 30,000 g for 15 min. The permeabilized mitochondria were resuspended in ice-cold 10 mM HEPES, 0.25 M sucrose and 0.2 mM EGTA, pH 7.4 at 4°C, and stored on ice until use. Protein was redetermined using the same method.

### Mitochondrial PDHC and OGDHC activity

NADH fluorescence was measured according to [51] in a 96-well Pherastar microplate reader at λ_excitation_ = 340 nm, λ_emission_ = 460 nm. Permeabilized mitochondria (0.1 mg mitochondrial protein/ml) were suspended at 37°C in 180 μl of 3 mM HEPES, 120 mM KCl, 1 mM EGTA, 5 mM KH_2_PO_4_, 2 mM MgCl_2_, 0.3% (w/v) fatty acid-free bovine serum albumin, pH 7.4 at 37°C, containing 0.3 mM ThDP, 0.9 mM CaCl_2_, 4 μM rotenone, 0.2 mM NAD^+^ and 0.14 mM CoASH. Total Ca^2+^ values were calculated using the software MaxChelator [52] to give 10 μM targeted free Ca^2+^. Reaction was started by pipetting the suspension described above into a plate previously loaded with different concentrations of pyruvate and pyruvate analogs shown in Fig. 4. OGDHC-dependent NADH production was measured titrating different concentrations of 2-oxoglutarate (0.05, 0.1, 0.2, 0.5 1 mM) over the same concentration of AcPH and AcPMe as in the PDH reaction (Fig. 4). Data on inhibition by AcPH and AcPMe were obtained using two separate mitochondrial preparations, with each activity point corresponding to a mean ± SEM of 3–6 technical replicates. There was a lag of ~ 0.5–1 min between loading the plate and starting the run. After that, the rate over the first minute was used to calculate the rate of NADH reduction. In control wells (without inhibitors) all NAD^+^ was reduced to NADH after 15–20 min, and the difference between initial and final fluorescence was used to calibrate the scale.

### Cellular studies

HEK293 and human glioblastoma cell lines LN405, T98G, U87 were obtained from the American Type Culture collection (LGC Standards GmbH; Wesel, Germany). Cells at a density of 2.5 × 10^4^ cells/ml, 200 μl per well, were seeded on microplates (Greiner, μClear, black clear bottom) in DMEM (4.5 g/L glucose, 10% FCS, 2 mM Glutamax, containing antibiotics). Medium was exchanged 24 h later for 100 μl per well of Hanks’ solution (HBSS) (1 g/L glucose, 0.37 M NaCl, 5.4 mM KCl, 0.25 mM Na_2_HPO_4_, 0.44 mM KH_2_PO_4_, 1.3 mM CaCl_2_, 1.0 mM MgSO_4_, 4.2 mM NaHCO_3_). *P*-analogs of pyruvate were added at different concentrations (0.2, 0.5, 1.0, 10 and 20 mM). 5 h later ATP levels were determined using the CellTiterGlo assay system (Promega, Heidelberg, Germany) according to manufacturer's recommendations as described previously [40]. Concentration dependence data were obtained by averaging luminescence from six wells, and % of the ATP levels in the treated vs control cells were used to characterize the effects of the analogs on cellular viability.

### Transcriptomics data analysis

To estimate the relative expression of different PDHC components, cellular monocarboxylate carriers (SLC16 family) and mitochondrial pyruvate carrier in relevant cells, we extracted the data on expression of proteins of interest from global mRNA expression experiments in the NCBI GEO database [53]. The expression in HEK293 cell line was from series GSE50547 (experiments GSM1221013, GSM1221014 and GSM1221015), GSE1822 (experiments GSM31805 and GSM31806), GSE1364 (experiments GSM22069, GSM22070 and GSM22071) and GSE1455 (experiments GSM24493, GSM24494 and GSM24495), in T98G cell line – from series GSE8537 (experiment GSM211868), GSE1692 (experiments GSM29233, GSM29234 and GSM29235) and GSE4218 (experiments GSM96274, GSM96275 and GSM96276) and in U87 cell line – from series GSE35169 (experiments GSM862922, GSM862923 and GSM862924, GSE9200 (experiment GSM231724), GSE1923 (experiments GSM34592, GSM34593 and GSM34594) and GSE53014 (experiments GSM1280363, GSM1280365, GSM1280374 and GSM1280376).

Fluorescence signals, which are background-corrected, scaled (normalized) and statistically analyzed by different algorithms (MAS 5.0, RMA or Limma), were extracted from the GEO database. Most of the data (series GSE50547, GSE1822, GSE8537, GSE1692, GSE35169, GSE9200 and GSE1923) were processed using the modern Affymetrix algorithm Microarray Suite version 5.0 (MAS 5.0). MAS 5.0 defines the signal for gene *i* as the anti-log of the robust average (Tukey's biweight) of Eq 1:

$$\text{log}(PM_{i,j}-CT_{i,j}),j=1\ldots J,$$ (1)

where PM_ij_ is the signal intensity values in the “perfect match” cells. CT_ij_ is defined as a quantity equal to “mismatch” (MM) values when *MM_ij_* < *PM_ij_*, but adjusted to be less then MM when *PM_ij_* » *PM_ij_*.

The Robust Multi-array Average (RMA) algorithm was used in older Affymetrix platforms. It does not use the “mismatch” values and summarizes the “perfect match” values using the median polish function. This method of data processing was used in series GSE1364, GSE1455 and GSE4218. Linear Models for Microarray Data (Limma) was used for processing the data from Agilent platforms (series GSE53014).

To compare different experiments, the processed fluorescence signals for the complete annotated genes of interest were extracted from GEO and normalized to the averaged mRNA levels of *GAPDH, ACTB* and *STAT1* in the same experiment. The median and SEM of these normalized values from *n* reported experiments were estimated.

### Metabolic profiling

Cells were grown on Petri dishes for 24 h. After changing the cellular growth medium to glucose-supplemented HBSS, 0.5 mM AcPH was added and cells were incubated for 5.5 h. Metabolic profiling was performed essentially as in [40]. Briefly, metabolites were extracted in 2 ml ice-cold methanol containing 0.05 mM ribitol as internal standard for the relative quantification of metabolite abundance [54]. After centrifugation, the supernatant was collected and stored frozen before the analyses. The pellet was used for protein quantification as in [55]. Samples were derivatized as in [54]. GC-MS metabolite determinations were normalized to ribitol level and protein content (μg). For the heat map visualisation normalized values were Log(2)-transformed and the heat map was created using MultiExperiment Viewer (MeV®) software [56].
